# Discovery of novel glioma serum biomarkers by proximity extension assay

**DOI:** 10.1186/s12014-023-09400-5

**Published:** 2023-03-24

**Authors:** Atefeh Ghorbani, Lisa M. Avery, Dorsa Sohaei, Andrea Soosaipillai, Maxime Richer, Craig Horbinski, Katy McCortney, Wei Xu, Eleftherios P. Diamandis, Ioannis Prassas

**Affiliations:** 1 Department of Laboratory Medicine and Pathobiology, University of Toronto, Toronto, Canada; 2 Biostatistics Division, Dalla Lana School of Public Health, University of Toronto, Toronto, Canada; 3 Department of Biostatistics, The Princess Margaret Cancer Centre/University of Toronto, Toronto, Canada; 4 Axe Neurosciences du Centre de Recherche du Centre Hospitalier Universitaire (CHU) de, Québec—Université Laval et Département de Biologie Moléculaire, Biochimie et Pathologie de l’Université, Laval, Québec Canada; 5 Feinberg School of Medicine, Northwestern Medicine Malnati Brain Tumor Institute of the Robert H. Lurie Comprehensive Cancer Center, Northwestern University, Chicago, IL USA; 6 Department of Pathology and Laboratory Medicine, Mount Sinai Hospital, Scientific Associate, Lunenfeld-Tanenbaum Research Institute, Toronto, ON Canada; 7 Lunenfeld-Tanenbaum Research Institute, Mount Sinai Hospital, Toronto, Canada; 8 Department of Clinical Biochemistry, University Health Network, Toronto, Canada; 9 Medical Biochemist, Mount Sinai Hospital and University Health Network Professor, Department of Laboratory Medicine & Pathobiology, University of Toronto, ACDC Lab, Room L6-201, 60 Murray St., Toronto, ON M5T 3L9 Canada

**Keywords:** Biomarkers, Early diagnosis, Glioma, Liquid biopsy, OLINK technology, Proximity extension assay (PEA)

## Abstract

**Background:**

Gliomas are among the most malignant tumors, with a very poor prognosis. Early diagnosis is highly desirable since it can help implement more effective treatments for smaller tumors, which have not yet extensively metastasized. Improving early diagnosis may facilitate access of patients to clinical trials and prepare them for the future availability of new disease-modifying treatments.

**Methods:**

We analyzed retrospective samples collected at diagnosis (before therapy initiation), with PEA (Olink Proteomics), quantifying about 3000 proteins. We utilized 30 plasmas from gliomas (20 glioblastomas, 5 anaplastic astrocytomas, 5 anaplastic oligodendrogliomas) and 20 meningiomas (as controls). We then analyzed the data to identify proteins which either alone, or in combination, could discriminate gliomas from meningiomas, or correlate with clinical and molecular alterations.

**Results:**

We identified 8 plasma proteins which were increased in gliomas vs. meningiomas (GFAP, NEFL, EDDM3B, PROK1, MMP3, CTRL, GP2, SPINT3) and 4 proteins which were decreased in gliomas vs. meningiomas (FABP4, ALDH3A1, IL-12B and OXT). Partition algorithms and logistic regression algorithms with two biomarkers (GFAP and FABP4) achieved sensitivity of 83% and 93% at 100% and 90% specificity, respectively. The strongest single marker was GFAP with an area under the ROC curve (AUC) of 0.86. The AUC for the GFAP-FABP4 combination was 0.98.

**Conclusion:**

PEA is a powerful new proteomic technology for biomarker discovery. GFAP and a handful of other plasma biomarkers may be useful for early glioma detection and probably, prognosis.

**Statement:**

Detecting gliomas as early as possible is highly desirable since it can significantly improve the chances of effective treatments. Reliable glioma biomarkers can timely inform glioma patients about the efficacy of their prescribed treatment. Our results reveal some novel putative glioma markers that may prove valuable, when used alone or in combination, towards improved clinical care of gliomas. In order to better appreciate the potential usefulness of these markers, their performance needs to be further validated in a larger cohort of samples.

## Introduction

Gliomas are tumors that originate in the glial cells of the brain or spine [1, 2]. Gliomas comprise about 30% of all brain tumors and 80% are malignant. Symptoms of gliomas depend on which part of the central nervous system is affected. A brain glioma can cause headaches, vomiting, and seizures. Gliomas do not usually metastasize through the blood circulation but they can spread via the cerebrospinal fluid.

High-grade gliomas are highly vascular tumors and tend to infiltrate diffusely. They usually have extensive areas of necrosis and hypoxia. Tumor growth can cause a breakdown of the blood-brain barrier in the vicinity of the tumor. As a rule, high-grade gliomas almost always recur, even after complete surgical excision. Low-grade gliomas grow slowly, often over many years, and can be followed without treatment unless they cause symptoms.

Gliomas are named according to the specific type of cell with which they share histological features, but not necessarily from which cell type they originate. The main types of gliomas are ependymomas (ependymal cells) [2] and astrocytomas (astrocytes). Glioblastoma multiforme is a malignant astrocytoma and the most common primary brain tumor among adults [1]. Oligodendrogliomas originate from oligodendrocytes. Mixed gliomas, such as oligoastrocytomas, contain cells from different types of glia.

Gliomas are further categorized according to their grade, which is determined by histopathologic evaluation of the tumor. The neuropathological evaluation of brain tumor specimens is usually performed according to the WHO classification of tumors of the central nervous system, briefly as follows [3].

- 1. Biologically benign gliomas [WHO grade 1] are comparatively low risk and can be removed surgically depending on their location.
- 2. Low-grade gliomas [WHO grade 2] are well-differentiated (not anaplastic). These tumors tend to exhibit benign tendencies and afford a better prognosis. However, they have a uniform rate of recurrence and increase in grade over time, so they are classified as malignant.
- 3. High-grade gliomas [WHO grades 3–4] are undifferentiated or anaplastic; these are malignant and carry a poor prognosis.

Treatment for brain gliomas depends on the location, the cell type, and the grade of malignancy. Often, treatment combines surgery, radiation therapy and chemotherapy. A prolonged survival was observed when treating with radiotherapy and concomitant temozolomide [4]. Treatment with contemporary immunotherapy may help with some gliomas [5].

Patients with gliomas carrying mutations in either IDH1 or IDH2 enzymes have a relatively favorable survival, compared with patients with gliomas with wild-type IDH1/2 genes [6]. In WHO grade 3 glioma, IDH1/2-mutated gliomas have a median survival of ~ 3.5 years, whereas IDH1/2 wild-type gliomas have a median overall survival of 1.5 years.

Apart from the major clinical unmet need for gliomas, which is development of safe and effective new therapeutics, a number of other advances may also help the patients with gliomas. These include:

- 1. Non-invasive earlier detection of brain tumors (or ruling-out glioma diagnosis), preferably with a simple blood test (liquid biopsy), in patients with symptoms, before imaging and biopsy is instituted. This may allow triaging of patients and reduction of expensive imaging costs.
- 2. Use of non-invasive tests for patient prognosis and monitoring treatment success or failure. This will become a major issue for therapy personalization and optimization, as new therapies become available.
- 3. Differential diagnosis of various brain tumor types and their grade without the need for biopsy, which requires craniotomy. Especially, blood-based biomarkers can be used to identify metastatic tumors to the brain from unknown primaries, that look similar to primary brain tumors on imaging.

In this paper we used for the first time, new and revolutionary technology (proximity extension assay; PEA; Olink Proteomics, Waltham, MA, USA) to identify novel serum biomarkers of brain tumors in a small cohort of patients, as a proof-of-principle. These candidate biomarkers, which can non-invasively be quantified in plasma, need further validation with larger sets of samples before they are brought closer to the clinic.

## Materials and methods

### Study population, sample collection and analysis

We used the Olink Proximity Extension Assay (PEA) to explore panel of 3000 proteins (Olink Proteomics, Waltham, MA, USA) to analyze a cohort of the following plasmas, provided by the Northwestern University Brain Tumor Biobank: 20 glioblastomas, 5 anaplastic astrocytomas, 5 anaplastic oligodendrogliomas and 20 meningiomas (as controls). All analyses were performed at Olink Proteomics facilities using PEA described in detail elsewhere [7, 8]. The list of 3000 proteins can be found on the Olink Website (info@olink.com). We previously validated PEA and found it to be reproducible, with CVs of < 20% for > 99% of the proteins [8]. After analysis, Olink provided an excel file consisting of 50 patients (plasma samples) with 3000 quantified proteins per sample (approximately 150,000 data points).

Some clinical information of the study participants is provided in (Table 1).

**Table 1:** Some demographic and clinical information of study participants

	Glioma	Meningioma
Participants, n	30	20
Age at sampling, in years	21–83	21–83
Sex-female, n (%)	7	14
Sex-male, n (%)	23	3

We used the following preliminary steps to prepare the data for analysis.

1. Protein abundances below the limit of detection (LOD) were set to the LOD for each protein.
2. After inspecting the distribution of abundances for each protein, log2 transformations were applied to normalize the data
3. Each protein reliability as a biomarker was tested by randomly allocating participants to two groups, stratified by disease type (glioma vs meningioma) and checking for statistical differences. Proteins exhibiting statistical differences in random groups were considered unreliable (note that no proteins were removed via this criterion).
4. Additional checks revealed that some proteins were repeated within panels; these were removed.

For all statistical analyses, all glioma sub-types were included in a single glioma group due to the small number of patients in each sub-group.

### Candidate biomarker selection

Volcano plots were created by calculating the difference in mean log-transformed abundance values between glioma and meningioma (log2 fold change) and plotting these log fold changes against p-values from t-tests comparing the mean differences in the log-transformed abundance values. Proteins with the greatest up-regulation for glioma are plotted furthest to the right and those with the greatest down-regulation furthest to the left. Proteins with fold-changes > 1 and p-values < 0.05 were highlighted. Given the exploratory nature of these analyses, no adjustment was made for multiple testing. In addition, proteins were screened using a non-parametric Wilcoxon rank sum test with a false discovery rate of 10%, in order to capture any additional proteins that may have large differences in median, but not mean values.

### Additional statistical analyses

Proteins identified in the candidate selection stage were further limited to those with FDR-corrected p-values of < 0.001, to build a model to predict disease type (glioma vs meningioma). A partitioning algorithm, developed at perfect specificity, was used to identify proteins predictive of glioma. This algorithm searches for the protein with the greatest sensitivity, conditional on achieving 100% specificity. Partitioning continues in this manner on the remaining participants until the marginal sensitivity of identifying the remaining participants falls below 50%. The final model describes cut-points for each protein that achieve perfect specificity.

A multivariable logistic regression model was fit to predict disease group from age and sex, to determine the importance of these clinical factors and univariate plots are provided to facilitate interpretation.

Univariate logistic regression analyses were run for each candidate biomarker and receiver operating characteristic (ROC) curves were constructed. The area under the curve (AUC) was calculated, along with 95% confidence intervals, using bootstrap resampling with 2000 bootstraps. A ROC curve was also constructed for the combination of GFAP and FABP4 from the logistic regression model.

### Survival analysis

There have only been five recorded deaths in this cohort at the time of analysis, so the sample is not well-powered to examine differences in survival status. For survival analysis, the seven cases with unknown survival status were treated as censored at the last known date of survival, similar to those known to have survived. Kaplan-Meier survival curves were then constructed to show the effect of each of the recorded molecular alterations on patient survival.

### Examining the effect of GFAP concentration on overall survival

A stratified Cox proportional hazard model was used to determine the risk of death associated with increased GFAP level, controlling for age and stratifying by diagnosis (glioma vs meningioma) to control for confounding.

## Results

### Volcano plot

**Fig. 1:**
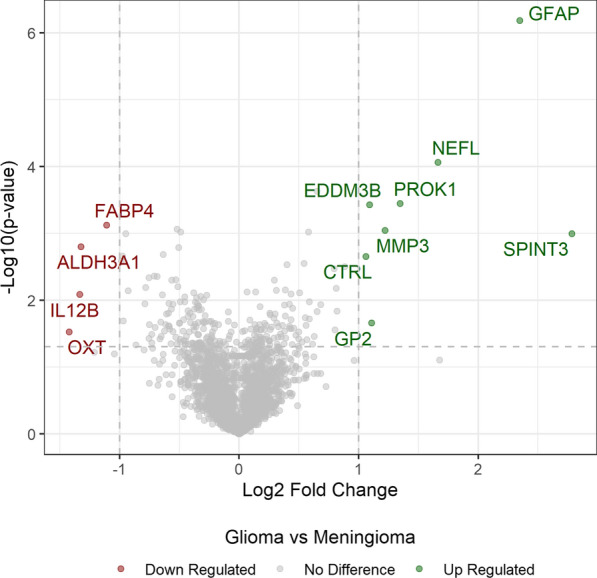
Volcano plots showing 8 up-regulated (green font) and 4 down-regulated (red font) proteins. For more details and discussion, see text. GFAP was the most up-regulated protein. The horizontal dashed line shows the cut-off p value

Up-regulated or down-regulated proteins refer to proteins that were increased (green font) or decreased (red font) in plasma of glioma vs meningioma patients (Fig. 1). We identified eight up-regulated and four down-regulated proteins, with GFAP displaying the most pronounced up-regulation. Table 1 presents the AUC for these 12, and three additional proteins: LMOD1, IDO1 and IL-8 (CXCL-8) which have previously been linked to glioma.

**Fig. 2:**
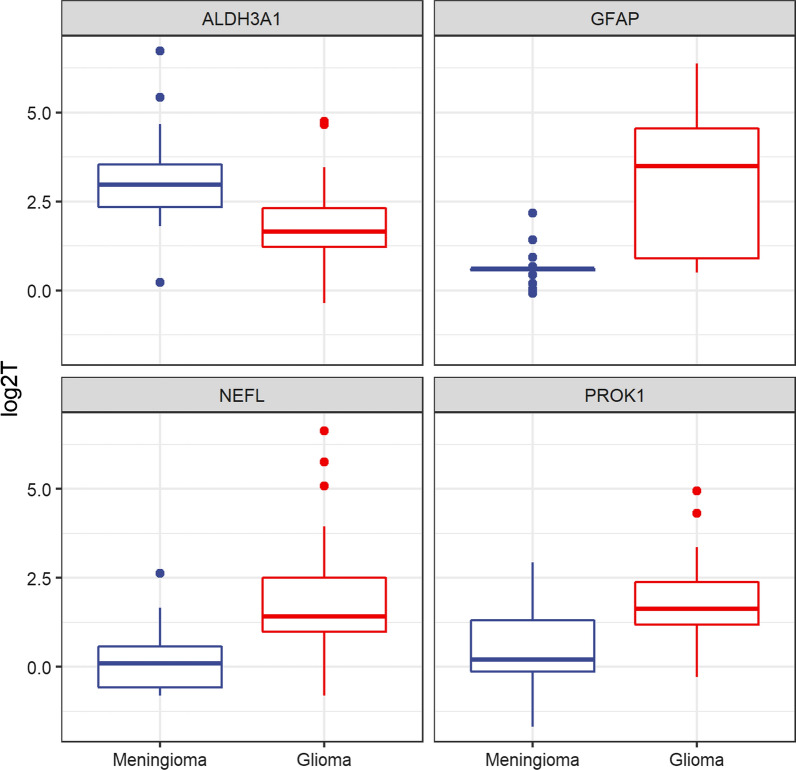
Box-plots of four representative proteins. One protein is down-regulated in gliomas (ALDH3A1) and 3 are up-regulated in gliomas (GFAP, NEFL and PROK1). Wilcoxon-Rank Sum p values were < 0.1 in all cases. For more details see text

Wilcoxon Rank Sum tests did not highlight any proteins in addition to the volcano plot. Examples of boxplots for four representative proteins (GFAP, ALDH3A1, NEFL and PROK1) are shown in (Fig. 2).

### Clinical predictors

**Fig. 3:**
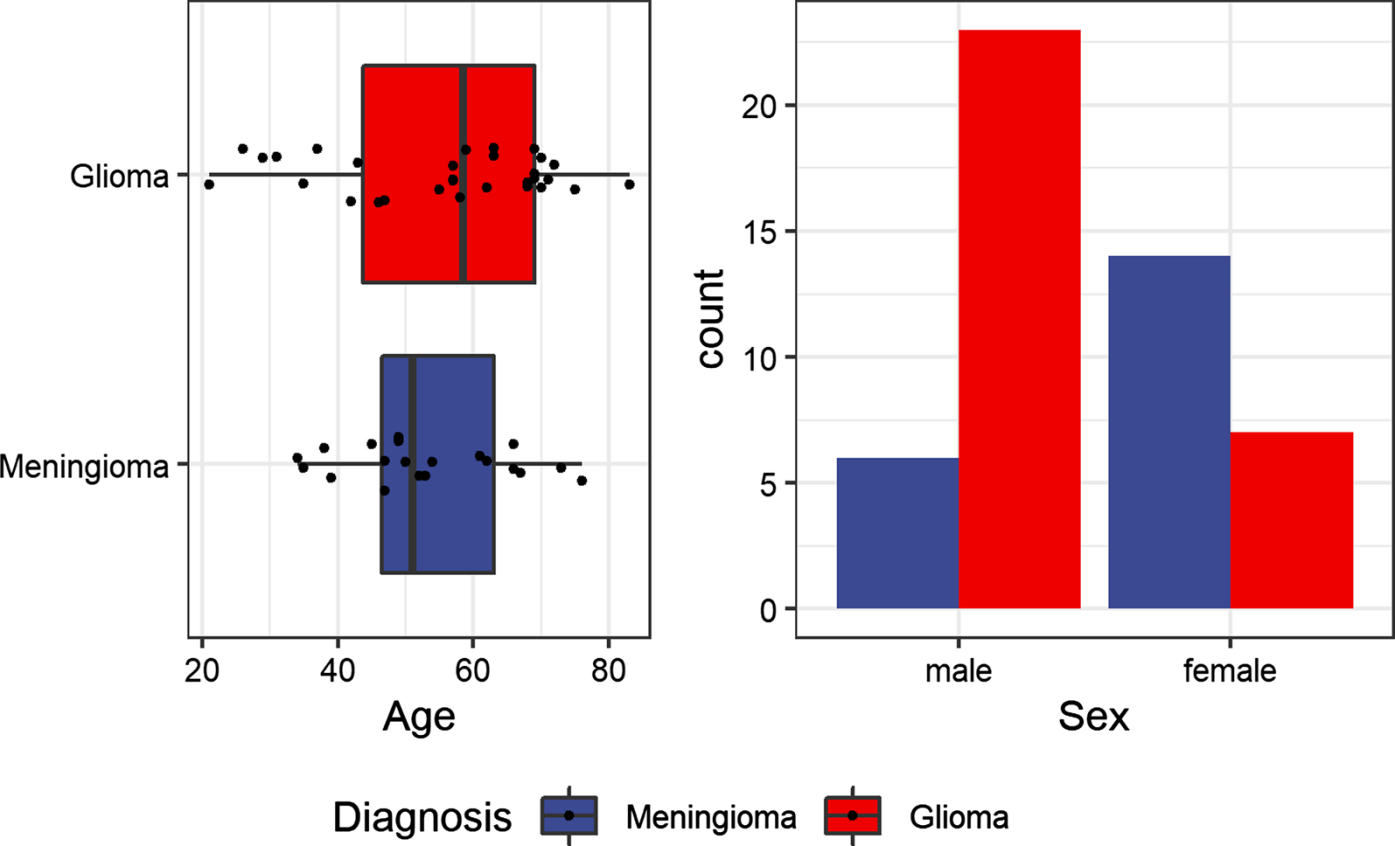
Age was not significantly different between patients with glioma or meningioma (left panel, p = 0.54) but glioma was most frequent in males than in females (p < 0.001). For comments see text

Multivariate logistic regressions were performed to determine if diagnosis was related to either patient age or sex. As expected, we found that sex, but not age, was associated with males more likely to have glioma and females more likely to have meningioma (Fig. 3).

### GFAP, FABP4 and other proteins

**Fig. 4:**
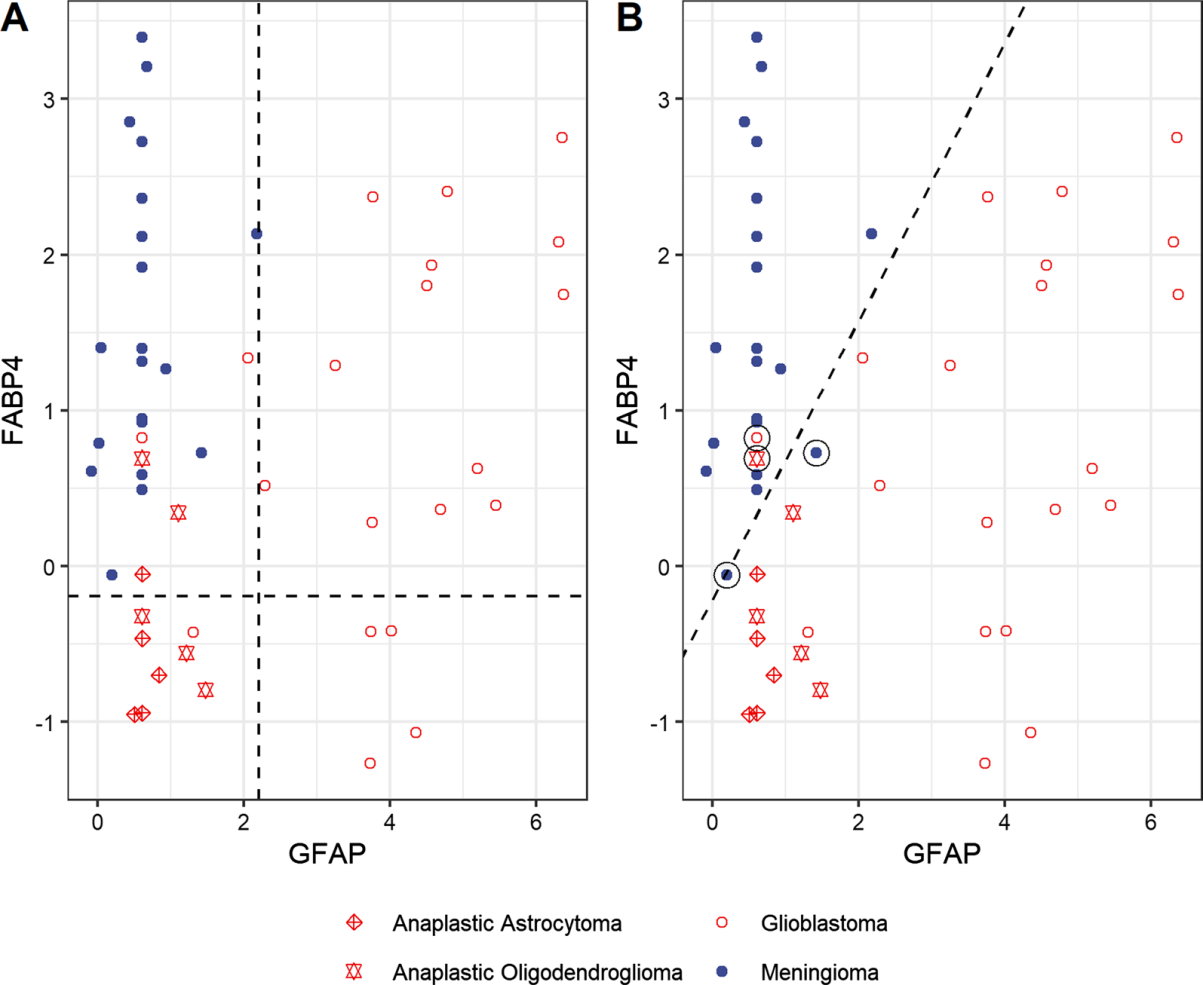
Combination of GFAP and FABP4 for discriminating gliomas (red circles) from meningiomas (blue dots) Anaplastic astrocytomas (red crossed diamonds) and anaplastic oligodendrogliomas (red double diamonds) are also shown. **A**: The vertical dashed line shows GFAP cut-ff at 100% specificity for gliomas and the horizontal dashed line shows FABP4 cut-off at 100% specificity for meningiomas. This model also aggregates the glioma cases by sub-type with glioblastomas having high GFAP, meningioma having low GFAP and high FABP4, while anaplastic astrocytomas and oligodendrogliomas display both low FABP4 and low GFAP [9, 10]. At 100% specificity (correct prediction of all 20 meningiomas) the sensitivity (correct prediction of gliomas) was 25/30 or 83%. **B**: Classification using logistic regression. We correctly classified 18/20 meningiomas (specificity of 90%) and 28/30 gliomas (sensitivity of 93%). The misclassified cases (two gliomas and two meningiomas) are circled

GFAP was the strongest predictor of disease status. Candidate proteins were plotted against GFAP to determine if there were proteins that could potentially improve GFAP’s prediction. Prediction was improved the most, by combining GFAP with FABP4. Interestingly, this model also aggregates the glioma cases by sub-type, with glioblastomas having high GFAP, meningiomas having low GFAP and high FABP4 [9, 10], while anaplastic astrocytomas and oligodendrogliomas display both low FABP4 and low GFAP (Fig. 4A). At 100% specificity (correct prediction of all 20 meningiomas) the sensitivity (correct prediction of gliomas) was 25/30 or 83%.

### Logistic regression

We fit a logistic regression model with GFAP and FABP4. The prediction was slightly better but at the expense of specificity. More specifically, we correctly classified 18/20 meningiomas (specificity of 90%) and 28/30 gliomas (sensitivity of 93%). (Fig. 4B) illustrates the classification via logistic regression, with cases to the right of line predicted to be gliomas. The four misclassified cases are circled.

**Fig. 5:**
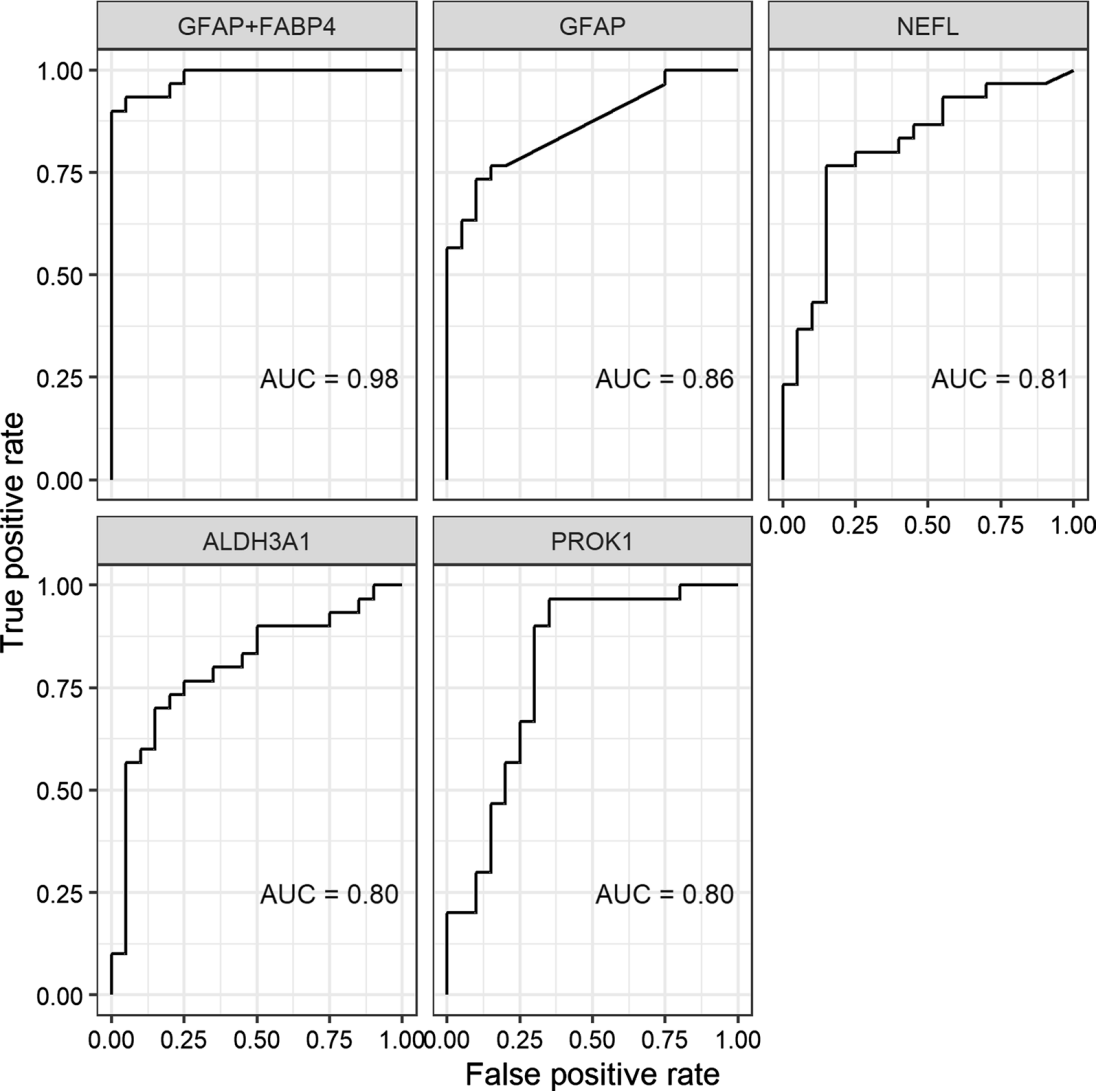
ROC curve for the combination of GFAP and FABP4 by logistic regression and ROC curves for the best four candidate biomarkers. The AUCs for all identified candidates are shown in Table 1. For discussion see text

Univariate logistic regression analyses were run for each candidate biomarker and receiver operating characteristic (ROC) curves were created. The area under the curve (AUC) was calculated, along with 95% confidence intervals. A ROC curve was also constructed for the combination of GFAP and FABP4 from the logistic regression model. (Fig. 5) shows the respective ROC curves. GFAP alone had an AUC of 0.86 and the GFAP-FABP4 combination had an AUC of 0.98. The AUCs and confidence intervals of all identified candidate biomarkers are shown in (Table 2).

**Table 2:** Areas under the curve (AUC) and 95% confidence intervals (CI) for all identified biomarkers in this study^1^

Biomarker	AUC	CI
GFAP	0.86	0.76, 0.95
NEFL	0.81	0.68, 0.92
PROK1	0.80	0.67, 0.92
ALDH3A1	0.80	0.66, 0.91
EDDM3B	0.78	0.64, 0.90
SPINT3	0.77	0.62, 0.90
FABP4	0.77	0.63, 0.88
CTRL	0.74	0.59, 0.87
MMP3	0.73	0.58, 0.86
OXT	0.69	0.52, 0.85
GP2	0.68	0.53, 0.82
IL12B	0.66	0.50, 0.80
LMOD1	0.57	0.41, 0.73
CXCL8	0.52	0.36, 0.69
IDO1	0.46	0.29, 0.64

*AUC* Area under the ROC Curve, *CI* 95% confidence intervals

### Survival analysis according to GFAP levels

**Fig. 6:**
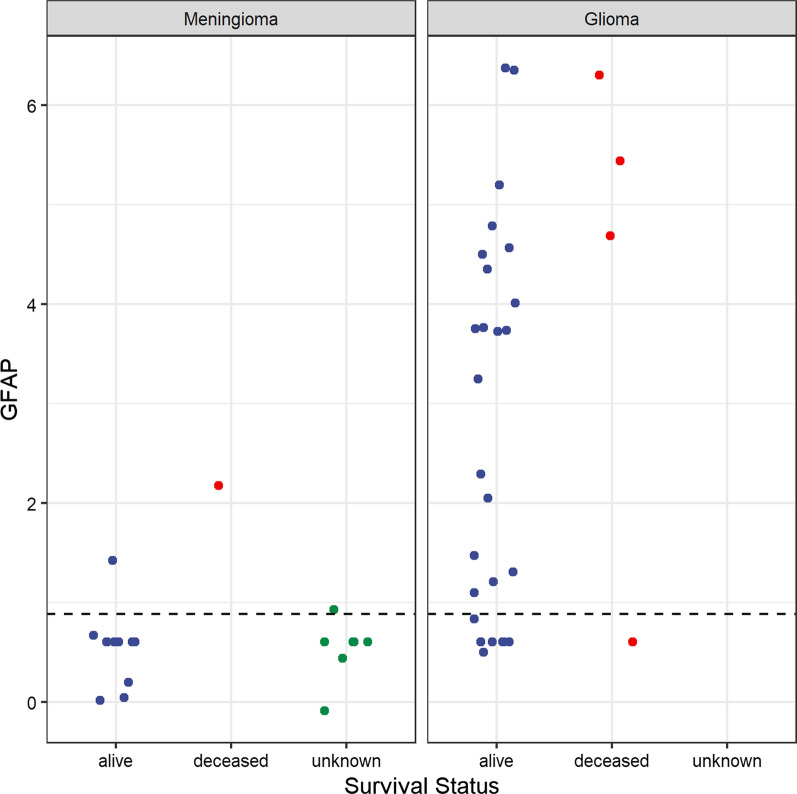
This plot shows the log-GFAP intensities for the diagnostic groups, colored by their survival status. The horizontal line is the median GFAP concentration. For comments see text

Figure 6 shows the log-GFAP intensities for the diagnostic groups, according to their survival status. The horizontal line is the median GFAP, which is highly correlated with disease type. A multivariable Cox model [11] controlling for age indicates a non-significant increased risk of death with higher GFAP levels. The hazard ratio (HR) and 95% confidence interval (CI) for high GFAP vs low GFAP was 1.61 (95% CI 0.71, 3.66; p = 0.26), while for age the HR was 0.98 (0.89, 1.09; p = 0.72).

### Effect of molecular changes on survival

**Fig. 7:**
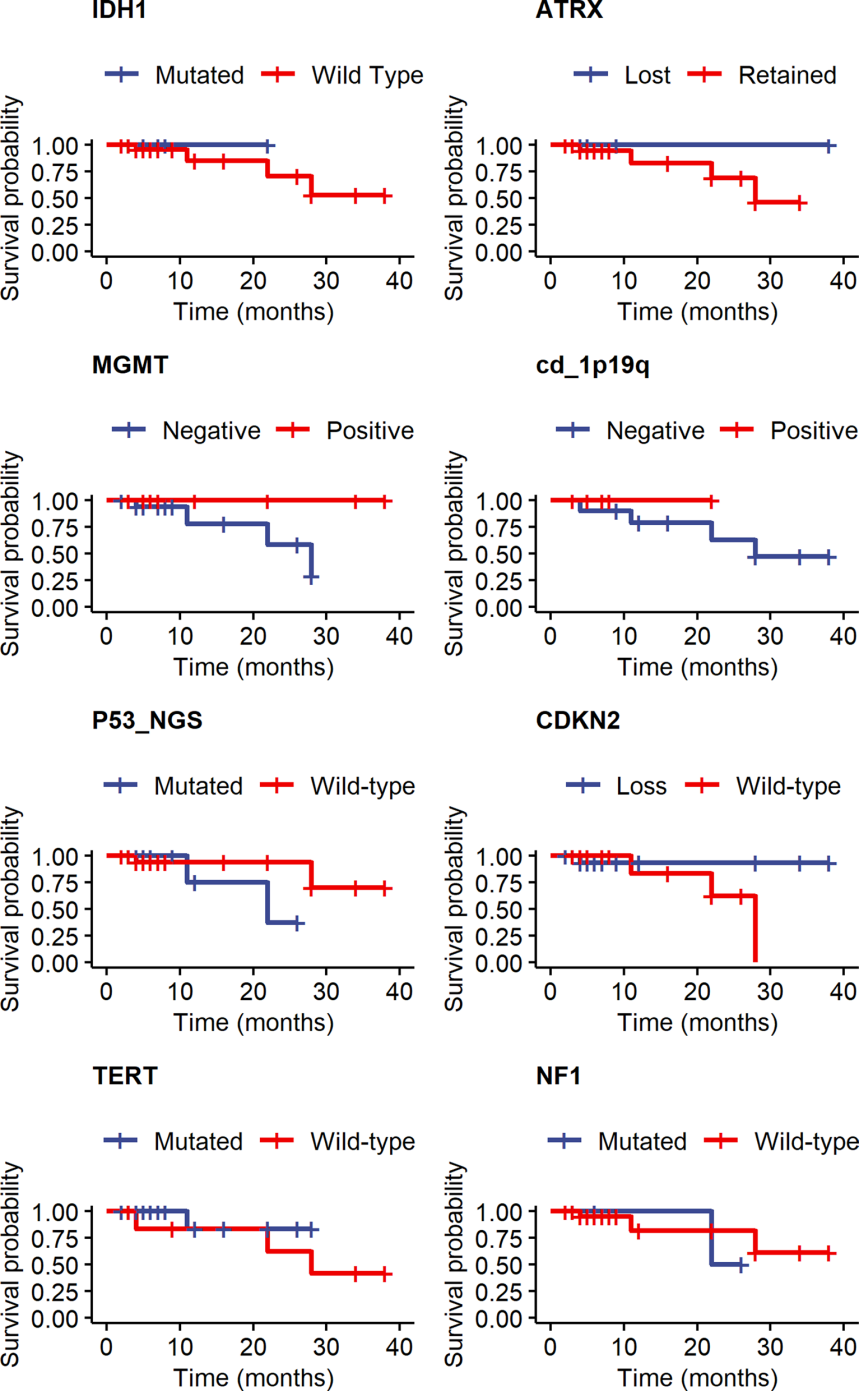
Kaplan-Meier survival curves of glioma patients according to molecular changes shown. For more discussion see text

Figure 7 shows Kaplan-Meier survival curves according to various molecular indices for gliomas. Due to the small number of cases and death events, statistical power was low, and p-values were not significant. However, the observed trends are in accord with what is known about patient survival and glioma molecular changes [6].

## Discussion

In this exploratory analysis, we used the highly sensitive PEA technology to analyze plasma samples collected from glioma patients before therapy and compared the results with age- and sex-matched control patients with meningiomas. The details of the PEA technology are described in our recent review [7]. Our laboratory has previously frequently used other proteomic technologies, such as mass spectrometry (MS) and ELISA for biomarker discovery [12–15] and we are in a good position to compare PEA, ELISA and MS technologies. Mass spectrometry multiplexing allows simultaneous analysis of 50–100 proteins by multiple reaction monitoring (MRM) [12], significantly less than PEA (> 3000 proteins measured in parallel). Also, MS suffers from low precision (CVs ~ 30–60%) for direct serum analysis. PEA precision is much better at 10–15%. Individual ELISA assays usually require 100 ul sample volume per assay (or, by extrapolation, 100 mL for 1000 assays, a volume which is unrealistic in clinical practice). PEA requires only 15 uL of sample for > 3000 assays. PEA is more specific than single ELISAs using the same antibodies due to dual recognition of both antibodies and nucleotides [7]. To summarize, PEA is a new, ultra-sensitive, highly specific and precise technology that requires no sample pre-treatment and can be highly multiplexed with minimal sample volume requirements. These properties qualify PEA as superior to LC/MS/MS and single ELISAs for liquid biopsy-based biomarker discovery applications.

To our knowledge, our work is the first demonstration of the use of this technology for glioma biomarker discovery. Most of the identified candidates have been shown in the past to have some relationship to gliomas/brain tumors, suggesting that our findings are unlikely to represent false discovery and are quite relevant to gliomas.

In this study, we found associations between glioma and several candidate plasma biomarkers. GFAP had the highest discriminatory potential between gliomas and meningiomas (Fig. 1), followed by several other candidates (Table 2).

The combination of just two markers (GFAP and FABP4) results in a ROC curve with an AUC of 0.98 (Fig. 5). Due to the small number of patients, these data should be interpreted with caution until they are independently validated with a larger number of patients.

For a long time, the structural role of GFAP in astrocytes [the main type of glial cells in the central nervous system (CNS)] was acknowledged [16]. More recently, GFAP has been demonstrated to be involved in numerous astrocyte functions. [17, 18]. After brain surgery, in the early stages of recovery, it has been found that GFAP increases in response to astrocytic reaction to the brain injury [19].

Recent emerging evidence supports the involvement of GFAP in glioblastoma multiforme (GBM). Serum GFAP was significantly increased in Grade 4 glioma and was detected in 63% of all Grade 4 patients compared to 13% of healthy controls, [20] indicating that glioma patients had higher GFAP levels, in accordance with our findings. [21, 22] Serum GFAP correlates with invasiveness and malignancy in astrocytomas and high-grade gliomas, compared to lower grade gliomas [16, 17]. Based on the Human Protein Atlas [23] GFAP is a highly brain-enriched protein (with very high expression in the central nervous system). Within the brain tissue, GFAP is expressed by the cerebral cortex, cerebellum, choroid plexus, basal ganglia, (hypo) thalamus, midbrain, pons, medulla oblongata, hippocampus, white matter, and amygdala, as well as in the spinal cord. High levels of GFAP are also seen in mouse brains. On the contrary FABP4, according to the same database [24] is mostly expressed by connective and soft tissues (mostly adipose tissues), followed by the Respiratory System and Female Tissues. Thus, GFAP is a potential biomarker and possible therapeutic target for gliomas.

Another well-known glioma biomarker is NEFL (Neurofilament light polypeptide) also known as neurofilament light chain, a potential tumor suppressor [25]. NEFL is involved in a variety of common human cancers such as breast, prostate, and head and neck cancers [26]. Serum NFL concentration was higher in patients with CNS tumors with disease in progression versus CNS tumors with stable disease [27]. In addition, serum NFL was higher in patients with metastatic solid tumors with known brain metastases than in those with metastatic tumors with no brain metastases [27].

Fatty acid-binding protein 4 (FABP4) is one of ten intracellular small molecular weight proteins that make up the FABP family [28, 29] and is found in adipose tissue, peripheral macrophages, and microglia [30]. Furthermore, it is not found in normal brain blood vessels, although it has been found in certain endothelial cells or tumor cells in benign and malignant meningiomas [10, 31]. FABP4 has a role in carcinogenesis in meningiomas by stimulating cell proliferation in a cell type-independent way. In this connection, rapamycin, a well-known inhibitor of the mTOR pathway, which is a master regulator of cell growth and metabolism, inhibits FABP4 production in endothelial cells [32]. FABP4 is expressed in a significantly higher percentage of GBMs in comparison to both normal brain tissues and lower-grade glial tumors. Data suggest that FABP4 may play a role in angiogenesis associated with GBMs**.** Another study analyzed FABP4 expression in a cohort of paraffin-embedded meningioma specimens by immunohistochemistry and double immunofluorescence analyses [9]. These results demonstrate that FABP4 is commonly expressed in meningioma vascular endothelial cells while tumor cell expression of FABP4 is primarily observed in anaplastic meningiomas. A combination of FABP4 immunostaining with histopathologic grading might provide a more accurate prediction of the biological behavior of meningiomas than histopathologic grading alone [31, 32].

MMP3 is involved in cell migration. PDGFR-alpha induces MMP3 gene expression and increased cell proliferation and cell migration upon stimulation by platelet-derived growth factor (PDGF). The induction of expression of MMP3 in glioblastoma cells triggers a cascade of gene expression, resulting in decreased cell adhesion and migration [33–35].

High plasma interleukin-8 (IL-8) was associated with short progression-free survival in newly diagnosed patients with Glioblastoma Multiforme (GBM). IL-8 was mostly secreted and expressed by mesenchymal GBM cell lines and expressed by vascular cells and immune cells. High plasma IL-8 at surgery was associated with short OS in newly diagnosed GBM [36].

Prokineticin 1 (PROK1) is a relatively conserved hypoxia-induced small protein mostly known for its ability to induce proliferation, migration and fenestration in the context of angiogenesis, pain perception and neurogenesis [37]. In a recent glioma biomarker discovery study, PROK1 expression in glioma tissue was found to be significantly higher than that in normal tissue (*P* < 0.05) with higher expression in high grade gliomas compared to low grade ones. Interestingly, glioma patients with higher PROK1 expression had a significantly shorter progression-free survival time further suggesting putative prognostic value of PROK1 in human gliomas [38].

Aldehyde dehydrogenase 3A1 (ALDH3A1) is a brain expressed (midbrain, hypothalamus) drug metabolizing protein that has been implicated in temozolomide-mediated resistance of glioma tumors [39]. Down-regulation of aldehyde dehydrogenase activity via Wnt/beta-catenin signaling blockade has been recently proposed as a possible means of reducing ALDH-mediated resistance to temozolomide (the chemotherapeutic drug currently used as standard treatment for glioblastoma) [40].

Interleukin 12 is a broad acting cytokine and a potent proinflammatory cytokine that is expressed by activated macrophages and acts as an essential inducer of Th1 responses. It is expressed in human brain (basal ganglia, hypothalamus, midbrain) and its expression in the context of glioma pathology it has been found to be mediated via insulin-like growth factor (IGF)-1 signaling networks [41]. Of note, a recent comprehensive transcriptome analysis of GBM microarray and data from The Cancer Genome Atlas (TCGA) database revealed interleukin 12 as a direct target certain microRNAs involved in GBM development [41].

Indoleamine 2, 3-dioxygenases 1 (IDO-1) is a tryptophan catabolizing enzyme that permits the conversion of tryptophan into kynurenine, a pathway with clear immunosuppressive functions. IDO1 targeting has emerged as a novel therapeutic opportunity in modern cancer immunotherapy [42]. Interestingly, while IDO1 is not generally expressed in the adult central nervous system, most of GBM patients do express significant levels of IDO1 [43]. Accumulating evidence highlight an important putative role for IDO1 in regulating tumor immunological escape in brain tumors [44, 45].

For the other identified candidate biomarkers (EDDM3B, LMOD1, GP2, SPINT3, CTRL, OXT) there is no specific literature linking them to gliomas or other brain tumors.

Similar to our own findings, the incidence of meningiomas is higher in females than males (Fig. 3) [31]. One of the reasons could be that in females, endogenous sex hormone levels are significantly higher during childbearing years [46]. Also, according to one of the previous studies, the risk of meningioma has increased because of reproductive variables or using exogenous sex hormones [47]. Also, according to previous studies, males have a higher incidence for glioma than females although this is not important in the pathological diagnosis and clinical treatment [48].

There are important limitations to this study, mainly due to the small number of patients. Consequently, these results need to be further validated in a larger cohort of samples. Specifically, the nature of the interaction between GFAP and FABP4 and its role in glioma diagnosis and prognosis requires further study.

## Conclusions

Overall, our results indicate that GFAP and a handful of other proteins could be valuable non-invasive biomarkers for glioma, but additional validation studies are needed. We anticipate that by using these biomarkers, alone or in combination, gliomas could be diagnosed and treated earlier, when the tumors are smaller, more sensitive to treatment, and have not yet widely metastasized.

## Data Availability

All raw data are securely stored in-house. Analyzed data are included at the manuscript.

## References

[1] Weller M, Wick W, Aldape K, Brada M, Berger M, Pfister SM (2015). Glioma. Nat Rev Dis Primers.

[2] Saleh AH, Samuel N, Juraschka K, Saleh MH, Taylor MD, Fehlings MG (2022). The biology of ependymomas and emerging novel therapies. Nat Rev Cancer.

[3] Komori T (2022). The 2021 WHO classification of tumors, 5th edition central nervous system tumors: the 10 basic principles. Brain Tumor Pathol.

[4] Omuro A, Brandes AA, Carpentier AF, Idbaih A, Reardon DA, Cloughesy T (2022). Radiotherapy combined with nivolumab or temozolomide for newly diagnosed glioblastoma with unmethylated MGMT promoter: an international randomized phase 3 trial. Neuro Oncol.

[5] Rong L, Li N, Zhang Z (2022). Emerging therapies for glioblastoma: current state and future directions. J Exp Clin Cancer Res.

[6] Richardson LG, Miller JJ, Kitagawa Y, Wakimoto H, Choi BD, Curry WT (2022). Implications of IDH mutations on immunotherapeutic strategies for malignant glioma. Neurosurg Focus.

[7] Ren AH, Diamandis EP, Kulasingam V (2021). Uncovering the depths of the human proteome: antibody-based technologies for ultrasensitive multiplexed protein detection and quantification. Mol Cell Proteomics.

[8] Ren A, Prassas I, Sugumar V, Soosaipillai A, Bernardini M, Diamandis EP (2021). Comparison of two multiplexed technologies for profiling >1000 serum proteins that may associate with tumor burden. F1000Res.

[9] Cataltepe O, Arikan MC, Ghelfi E, Karaaslan C, Ozsurekci Y, Dresser K (2021). Fatty acid binding protein 4 is expressed in distinct endothelial and non-endothelial cell populations in glioblastoma. Neuropathol Appl Neurobiol.

[10] Lee V, Smith TW, Arikan MÇ, Zhang L, Çataltepe O, Çataltepe S (2021). Fatty acid-binding protein 4 expression in tumor cells as a potential marker for anaplastic meningiomas. Appl Immunohistochem Mol Morphol.

[11] Abd ElHafeez S, D'Arrigo G, Leonardis D, Fusaro M, Tripepi G, Roumeliotis S (2021). Methods to analyze time-to-event data: the cox regression analysis. Oxid Med Cell Longev.

[12] Begcevic I, Brinc D, Dukic L, Simundic AM, Zavoreo I, Basic Kes V (2018). Targeted mass spectrometry-based assays for relative quantification of 30 brain-related proteins and their clinical applications. J Proteome Res.

[13] Kulasingam V, Pavlou MP, Diamandis EP (2010). Integrating high-throughput technologies in the quest for effective biomarkers for ovarian cancer. Nat Rev Cancer.

[14] Kuk C, Kulasingam V, Gunawardana CG, Smith CR, Batruch I, Diamandis EP (2009). Mining the ovarian cancer ascites proteome for potential ovarian cancer biomarkers. Mol Cell Proteomics.

[15] Diamandis EP (2004). Mass spectrometry as a diagnostic and a cancer biomarker discovery tool: opportunities and potential limitations. Mol Cell Proteomics.

[16] Bignami A, Eng LF, Dahl D, Uyeda CT (1972). Localization of the glial fibrillary acidic protein in astrocytes by immunofluorescence. Brain Res.

[17] Middeldorp J, Hol EM (2011). GFAP in health and disease. Prog in Neurobiol.

[18] Hol EM, Pekny M (2015). Glial fibrillary acidic protein (GFAP) and the astrocyte intermediate filament system in diseases of the central nervous system. Curr Opin Cell Biol.

[19] Baumgarten P, Quick-Weller J, Gessler F, Wanger M, Tichy J, Froster MT (2018). Pre- and early postoperative GFAP serum levels in glioma and brain metastases. J Neurooncol.

[20] Van Asperen JV, Fedorushkova DM, Robe PAJT, Hol EM (2022). Investigation of glial fibrillary acidic protein (GFAP) in body fluids as a potential biomarker for glioma: a systematic review and meta-analysis. Biomarkers.

[21] Wei P, Zhang W, Yang LS, Zhang HS, Xu XE, Jiang YH (2013). Serum GFAP autoantibody as an ELISA-detectable glioma marker. Tumour Biol.

[22] Kiviniemi A, Gardberg M, Frantzen J, Parkkola R, Vuorinen V, Pesola M (2015). Serum levels of GFAP and EGFR in primary and recurrent high-grade gliomas: correlation to tumor volume, molecular markers, and progression-free survival. J Neurooncol.

[23] The Human Protein Atlas. 2023. https://www.proteinatlas.org/ENSG00000131095-GFAP/tissue. Accessed 15 February 2023.

[24] The Human Protein Atlas. 2023. https://www.proteinatlas.org/ENSG00000170323-FABP4/tissue. Accessed 15 February 2023.

[25] Wang ZY, Xiong J, Zhang SS, Wang JJ, Gong ZY, Dia MH (2016). Up-regulation of microRNA-183 promotes cell proliferation and invasion in glioma by directly targeting NEFL. Cell Mol Neurobiol.

[26] Chen B, Chen J, House MG, Cullen KJ, Nephew KP, Gou Z (2012). Role of neurofilament light polypeptide in head and neck cancer chemoresistance. Mol Cancer Res.

[27] Hepner A, Porter J, Hare F, Nasir SS, Zetterberg H, Blennow K (2019). Serum neurofilament light, glial fibrillary acidic protein and tau are possible serum biomarkers for activity of brain metastases and gliomas. World J Oncol.

[28] Li HY, Lv BB, Bi YH (2018). FABP4 accelerates glioblastoma cell growth and metastasis through Wnt10b signalling. Eur Rev Med Pharmacol Sci.

[29] Hertzel AV, Bernlohr DA (2000). The mammalian fatty acid-binding protein multigene family: molecular and genetic insights into function. Trends Endocrinol Metab.

[30] Duffy CM, Xu H, Nixon JP, Bernlohr DA, Butterick TA (2017). Identification of a fatty acid binding protein4-UCP2 axis regulating microglial mediated neuroinflammation. Mol Cell Neurosci.

[31] Lee V, Smith TW, Arikan MC, Zhang L, Cataltepe O, Cataltepe S (2021). Fatty acid-binding protein 4 expression in tumor cells as a potential marker for anaplastic meningiomas. Appl Immunohistochem Mol Morphol.

[32] Elmasri H, Ghelfi E, Yu CW, Traphagen S, Cernadas M, Cao H (2012). Endothelial cell-fatty acid binding protein 4 promotes angiogenesis: role of stem cell factor/c-kit pathway. Angiogenesis.

[33] Li L, Du Y, Xiang D, Chen L, Shi Z, Tian J (2018). Prediction of the anti-glioma therapeutic effects of temozolomide through in vivo molecular imaging of MMP expression. Biomed Opt Express.

[34] Yu X, Jin J, Zheng Y, Zhu H, Xu H, Ma J (2021). GBP5 drives malignancy of glioblastoma via the Src/ERK1/2/MMP3 pathway. Cell Death Dis.

[35] Laurent M, Martinerie C, Thibout H, Hoffman MP, Verrecchia F, Bouc YL (2003). NOVH increases MMP3 expression and cell migration in glioblastoma cells via a PDGFR-alpha-dependent mechanism. FASEB J.

[36] Holst CB, Christensen IJ, Vitting-Seerup K, Skjoth-Rasmussen J, Hamerlik P, Poulsen HS (2021). Plasma IL-8 and ICOSLG as prognostic biomarkers in glioblastoma. Neurooncol Adv.

[37] Zhao Y, Wu J, Wang X, Jia H, Chen DN, Li JD (2019). Prokineticins and their G protein-coupled receptors in health and disease. Prog Mol Biol Transl Sci.

[38] Xiao B, Tan L, Li D, Wang L, Xiao X, Meng G (2017). Clinical and prognostic significance of prokineticin 1 in human gliomas. Int J Clin Exp Pathol.

[39] Stavrinou P, Mavrogiorgou MC, Polyzoidis K, Kreft-Kerekes V, Timmer M, Marselos M (2015). Expression profile of genes related to drug metabolism in human brain tumors. PLoS ONE.

[40] Suwala AK, Koch K, Rios DH, Aretz P, Uhlmann C, Ogorek I (2018). Inhibition of Wnt/beta-catenin signaling downregulates expression of aldehyde dehydrogenase isoform 3A1 (ALDH3A1) to reduce resistance against temozolomide in glioblastoma in vitro. Oncotarget.

[41] Ho KH, Chen PH, His E, Shih CM, Chang WC, Cheng CH (2017). Identification of IGF-1-enhanced cytokine expressions targeted by miR-181d in glioblastomas via an integrative miRNA/mRNA regulatory network analysis. Sci Rep.

[42] Liu M, Wang X, Wang L, Ma X, Gong Z, Zhang S (2018). Targeting the IDO1 pathway in cancer: from bench to bedside. J Hematol Oncol.

[43] Hosseinalizadeh H, Mahmoodpour M, Samadani AA, Roudkenar MH (2022). The immunosuppressive role of indoleamine 2, 3-dioxygenase in glioblastoma: mechanism of action and immunotherapeutic strategies. Med Oncol.

[44] Agarwal P, Beale OM, Zhang X, Sandlesh P, Jaman E, Amankulor N (2022). Machine learning identification of immunotherapy targets in low-grade glioma using RNA sequencing expression data. World Neurosurg.

[45] Zhai L, Bell A, Ladomersky E, Lauing KL, Bollu L, Sosman JA (2020). Immunosuppressive IDO in cancer: mechanisms of action, animal models, and targeting strategies. Front Immunol.

[46] Hatch EE, Linet MS, Zhang J, Fine HA, Shapiro WR, Selker RG (2005). Reproductive and hormonal factors and risk of brain tumors in adult females. Int J Cancer.

[47] Korhonen K, Raitanen J, Isola J, Haapasalo H, Salminen T, Aubinen A (2010). Exogenous sex hormone use and risk of meningioma: a population-based case-control study in Finland. Cancer Causes Control.

[48] Inskip PD, Linet MS, Heineman EF (1995). Etiology of brain tumors in adults. Epidemiol Rev.

